# Sedation, analgesia, and *delirium* management in Portugal: a survey and point prevalence study

**DOI:** 10.5935/0103-507X.20220020-en

**Published:** 2022

**Authors:** Maria Carolina Paulino, Isabel Jesus Pereira, Vasco Costa, Aida Neves, Anabela Santos, Carla Margarida Teixeira, Isabel Coimbra, Paula Fernandes, Ricardo Bernardo, Pedro Póvoa, Cristina Granja

**Affiliations:** 1 Polivalent Intensive Care Unit, Hospital de São Francisco Xavier, Centro Hospitalar de Lisboa Ocidental - Lisboa, Portugal.; 2 Intensive Care Medicine Department, Centro Hospitalar de Gaia/Espinho - Vila Nova de Gaia, Portugal.; 3 Intensive Care Medicine Department, Centro Hospitalar Universitário de São João - Porto, Portugal.; 4 Intensive Care Medicine/Anesthesiology Service, Centro Hospitalar Trás-os-Montes - Alto Douro, Portugal; 5 Department of Anesthesiology and Intensive Care, Centro Hospitalar Universitário do Porto - Porto, Portugal.; 6 Department of Anesthesiology, Centro Hospitalar Universitário Lisboa Norte - Lisboa, Portugal.

**Keywords:** Analgesia, Sedation, Delirium, Critical care, Survey and questionnaires

## Abstract

**Objective:**

To establish current Portuguese critical care practices regarding analgesia, sedation, and *delirium* based on a comparison between the activities reported and daily clinical practice.

**Methods:**

A national survey was conducted among physicians invited to report their practice toward analgesia, sedation, and *delirium* in intensive care units. A point prevalence study was performed to analyze daily practices.

**Results:**

A total of 117 physicians answered the survey, and 192 patients were included in the point prevalence study. Survey and point prevalence studies reflect a high sedation assessment (92%; 88.5%), with the Richmond Agitated Sedation Scale being the most reported and used scale (41.7%; 58.2%) and propofol being the most reported and used medication (91.4%; 58.6%). Midazolam prescribing was reported by 68.4% of responders, but a point prevalence study revealed a use of 27.6%.

Although 46.4% of responders reported oversedation, this was actually documented in 32% of the patients. The survey reports the daily assessment of pain (92%) using standardized scales (71%). The same was identified in the point prevalence study, with 91.1% of analgesia assessment mainly with the Behavioral Pain Scale. In the survey, opioids were reported as the first analgesic. In clinical practice, acetaminophen was the first option (34.6%), followed by opioids. *Delirium* assessment was reported by 70% of physicians but was performed in less than 10% of the patients.

**Conclusion:**

The results from the survey did not accurately reflect the common practices in Portuguese intensive care units, as reported in the point prevalence study. Efforts should be made specifically to avoid oversedation and to promote delirium assessment.

## INTRODUCTION

Critically ill patients frequently require analgesia and sedation to facilitate distressing interventions, improve patient ventilator synchrony, relieve anxiety and improve comfort and safety. Eventually, some patients will develop *delirium*, known to be associated with cognitive impairment at 3 and 12 months after intensive care unit (ICU) discharge,^(1,2)^ longer hospital stays^(3)^ and higher mortality.^(4,5)^

Several validated scales have been recommended for the assessment of pain, agitation, and *delirium*.^(6)^ Pain treatment and light sedation are both associated with better outcomes^(7)^ and a reduction in *delirium* prevalence. Without adequate *delirium* assessment, there is a high risk of underestimating *delirium*, especially hypoactive *delirium*.^(8)^

To improve our practice, it is important to understand our current practices so that we can identify the best targets for improvement. Other surveys conducted in different countries analyzed their own specific practices concerning analgesia, sedation and *delirium* (ASD) in critically ill patients.^(9-16)^ All of them gave information about explicit targets for quality improvement in each specific country.

In the United Kingdom^(17)^ and Germany,^(18)^ studies with designs similar to ours were conducted, with two different evaluations. They compared the physicians’ analgesia, sedation and *delirium* practices reported in a survey with a subsequent daily clinical practice analysis. In both countries, the information reported in the survey did not accurately reflect the clinical practice observed.

The aim of this study was to characterize analgesia, sedation, and *delirium* practices in Portuguese ICUs, particularly the adherence to international recommendations, provide specific targets for improvement, and establish the priority for further research and national recommendations.

## METHODS

### National survey

We conducted a MEDLINE search using the keywords “analgesia”, “sedation”, “*delirium*” and “ICU” to identify the most important aspects in the literature regarding this area. The survey had five parts: 1) professional profile and ICU characterization (8 questions); 2) generic information about guideline application and follow-up (4 questions); 3) sedation practices (9 questions); 4) analgesia practices (4 questions); and 5) *delirium*, rehabilitation/mobilization and sleep improvement (17 questions) (Appendix 1S - Supplementary material). The questions referred to participants’ perceptions of their ICU daily practice. The format of the questionnaire was tick boxes and blank spaces for completion, with an average duration of 15 minutes, and questions were not mandatory. To ensure that the survey was robust, sensitive, and reliable, it was presented to the GASD (Analgesia, Sedation and *Delirium* Study Group). This group is a critical care research group that includes intensivists and ICU trainees who commented on, discussed and approved the final version.

Between 1 September 2016 and 30 April 2017, the survey was distributed through a link sent by e-mail to all ICU physicians registered in the Portuguese Intensive Care Society, irrespective of their working place being the National Health Service or a private setting. Neonatal and pediatric ICUs were excluded. The survey was designed using a web-based provider (MedQuest) accompanied by a cover letter, which informed the respondent of the details required for its completion.

The survey did not contain any data that could identify the respondents. Participation in the survey was anonymous, voluntary, and noninterventional.

To reinforce participation, the survey link was sent two times during this period. There was a limitation in the survey response; only the professionals who had not responded to the first link could open and fill the second survey link that was sent (to avoid duplicate answers).

### Point prevalence study

A point prevalence study (PPS) was performed on the 26^th^ of January 2018 in adult ICUs. We excluded high dependency units and cardiac and cardiothoracic ICUs. Invitations were sent to the directors of 20 ICUs in

Portugal from north to south, and 17 agreed to participate. The study was approved by the Research and Ethics Committee of *Centro Hospitalar de Lisboa Ocidental*. Each hospital received ethics approval from its local Institutional Review Board and was granted a waiver of informed consent for this observational, minimal risk study. No incentives were offered to participants. No financial rewards were granted to participating centers.

The participating ICU team was asked to complete a short data collection form (Appendix 2S - Supplementary material) for each patient (≥ 18 years) in the ICU between 00:00 and 24:00h on the 26^th^ of January 2018. The exclusion criteria were death during the study period and withholding or withdrawing life-sustaining treatment decisions.

In each ICU, there was one physician dedicated to conducting the study, checking the eligibility, and collecting all the data during the study day (24 hours). Most of the data were collected directly from the patients’ records; there was no intervention in clinical practice, no additional assessments, and no changes in the normal routine in the ICU. The collection form was returned on paper. All data were entered into a dedicated electronic database exclusively created for this study and managed by the authors who ensured its confidentiality. Patients were given a code number to secure their identification.

### Data and statistical analysis

Standard descriptive statistics were used as appropriate, and variables were reported as numbers (%). Continuous variables are expressed as the mean ± standard deviation, and categorical variables are expressed as n (%). As the number of respondents varied across the questions, with some missing answers, the proportions displayed in the results section and tables were not constant. Statistical analysis was performed using IBM SPSS Statistics v26.0 (IBM, Somers, NY, USA).

## RESULTS

### National survey

The overall response rate was 28% (117/418 physicians). The majority of respondents (91.2%; 103/113) worked exclusively in the ICU and specialized in intensive care medicine (68%; 78/114), with a mean age of 47 years and a mean of 12 years of ICU practice. The ICUs had a mean of 13 beds (5 beds level 2 and 10.7 beds level 3) and were predominantly distributed in the north and center of the country (84%), and most of them had a mixed medicalsurgical patient case mix (89.8%) (Table 1).

**Table 1:** Survey: characteristics of respondents

Number of surveys sent	418
Response rate	117 (28)
Sex (n = 115) Male	60 (52.2)
Female	55 (47.8)
Age (n = 115)	47.4 (±10.2)
Specialization in Intensive Care Medicine (n = 114) Yes	78 (68)
No	36 (32)
Number of years in ICU practice (n = 110)	12.35 (± 8.5)
Distribution of physicians in the country (n = 113) North	40 (35)
Center	55 (49)
South	13 (12)
Islands	5 (4)
ICU characterization (n = 118) Mixed	106 (89.8)
Surgical	6 (5)
Neurocritical	2 (1.7)
Medical	2 (1.7)
Cardiac	1 (0.8)
Burn patients	1 (0.8)
Number of ICU beds (n =108)	13.3 (± 7,6)
Invasive mechanical ventilation (n = 108)	70.2 (± 20.7)
Duration (days) (n = 98)	5.6 (2.5)

ICU - intensive care unit. The results are presented as n (%) or mean ± standard deviation.

The existence of protocols for ASD was considered useful by 95% (104/110) of the physicians, but less than 50% referred to having such protocols in their ICUs (Table 2).

**Table 2:** Survey analysis of protocols regarding analgesia, sedation and *delirium*

Do you consider protocols useful? (n = 110) Yes	104 (95)
No	6 (5)
Do you have protocols for analgesia, sedation or *delirium* in your ICU? (n = 111) Yes	55 (49.5)
No	56 (50.5)
Do you perform daily sedation monitoring? (n = 111) Yes	102 (92)
No	9 (8)
How do you describe the adequacy of sedation in your patients? (n = 112) Insufficient sedation	2 (1.8)
Appropriate sedation	58 (51.7)
Oversedation	52 (46.4)
How is sedation assessment done? (n = 234)^*^ RASS	98 (41.7)
RSS	40 (17.0)
Physician clinical evaluation	37 (15.7)
GCS	36 (15.3)
Nurse evaluation	20 (8.5)
MAAS	2 (0.9)
ATICE	1 (0.4)
Do you perform Spontaneous Awakening Trials? (n = 111) Yes	60 (54)
Não	51 (46)
Do you perform Spontaneous Breathing Trials? (n = 106) Yes	51 (48)
No	55 (52)
In what percentage of patients do you prescribe analgesia? (n = 109)	87.5% ± 15.6
Do you undertake daily analgesia assessment? (n = 109) Yes	101 (93)
No	8 (7)
Do you use any scale for pain assessment? (n = 106) Yes	75 (71)
No	31 (29)
Which scale(s) do you use for pain assessment? (n = 146)^*^ BPS	55 (37.7)
VAS	32 (21.9)
NRS	27 (18.5)
Faces scale	22 (15.0)
VRS	8 (5.5)
ESCID	1 (0.7)
CPOT	1 (0.7)
How frequently do you consider *delirium* in your ICU? (n=104)	37.3% ± 20.4
Do you do daily *delirium* assessment? (n = 110) Yes	77 (70)
No	33 (30)
How is *delirium* assessed? (n = 101) CAM-ICU	55 (54.5)
Clinical evaluation	42 (41.6)
ICDSC	4 (4.0)
Are *delirium* scales easy to apply? (n = 91) Yes	34 (37.4)
No	57 (62.6)

ICU - intensive care unit; RASS - Richmond Agitation-Sedation Scale; RSS - Ramsay Sedation Scale; GCS - Glasgow Coma Scale; MAAS - Motor Activity Assessment Scale; ATICE - Adaptation to Intensive Care Environment; BPS - Behavioral Pain Scale; VAS - Visual Analog Scales; NRS - Numeric Rating Scale; VRS - Verbal Rating Scales; ESCID - *Escala de Conductas Indicadoras de Dolor*; CPOT - Critical Care Pain Observation Toll; CAM-ICU - Confusion Assessment Method for Intensive Care Unit; ICDSC - Intensive Care Delirium Screening Checklist. The response rate was not uniform per question.

* Multiple option question. Results presented as n (%) or mean ± standard deviation.

### Sedation assessment

Concerning sedation, 92% (102/111) of the respondents reported daily sedation assessment. The most commonly used scales were Richmond Agitated Sedation Scale (RASS)^(19)^ 41.7% (98/235) and Ramsay Sedation Scale (RSS)^(6)^ 17% (40/235). Some physicians (15.7%, 37/235) reported the use of clinical evaluation without specific sedation assessment tools (Table 2).

Most of the respondents described doing such an assessment three times per day (40.2%; 47/117). Spontaneous awakening trials with daily sedation interruption were reported by 54% (60/111) of respondents.

**Figure 1:**
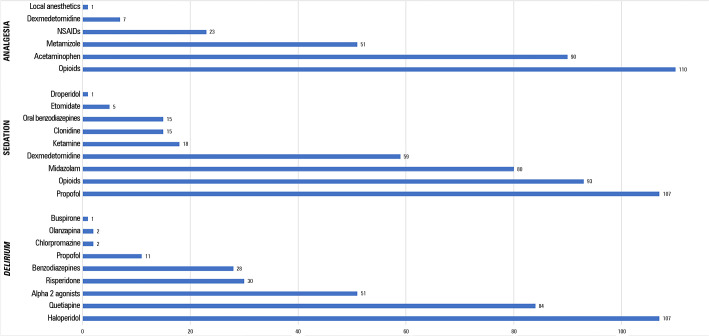
Survey: first-line therapy for pain management (n = 282), sedation (n = 393) and hyperactive delirium treatment (n = 316). NSAIDs - nonsteroidal anti-inflammatory drugs.

The most frequently used drugs were propofol (91.4% of physicians 107/117), followed by opioids (79%; 93/117), midazolam (68%; 80/117) and dexmedetomidine (50%; 59/117) (Figure 1).

Concerning the adequacy of sedation, 46% (52/112) considered that patients were oversedated most of the time, 2% (2/112) reported insufficient sedation, and 52% (58/112) considered sedation to be appropriate.

### Pain assessment

Pain was described as a frequent problem in the ICU (99%; 111/112), and 93% of the respondents (101/109) reported monitoring pain daily, but only 71% (75/106) adopted standardized scales. The Behavioral Pain Scale (BPS) and Visual Analog Scales (VAS) were the most commonly used scales (Table 2), and opioids were the most commonly used analgesic (94%; 110/117), followed by acetaminophen (77%; 90/117) (Figure 1).

Analgesia by regional blockade was reported by 67.6% of the physicians (75/111) as part of multimodal analgesia^(20,21)^ (use was reported in 94% (100/106)).

### *Delirium* assessment

Most physicians (70%; 77/110) reported performing daily *delirium* monitoring. The Confusion Assessment Method for Intensive Care Unit (CAMI CU)^(22)^ was the most reported *delirium* assessment tool (54.5%; 55/101), and the Intensive Care *Delirium* Screening Checklist (ICDSC)^(23)^ was reported by only 4% (4/101). Some physicians described *delirium* assessments based on clinical evaluation without the use of scales (41.6%; 42/101). Almost half of the respondents considered *delirium* scales easy to apply (37.4%; 34/91).

**Figure 2:**
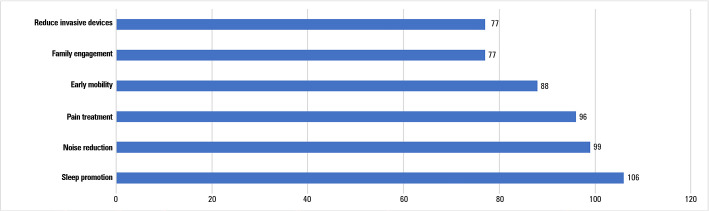
Nonpharmacological treatment for intensive care unit delirium (n = 543).

*Delirium* prevention and treatment therapies included both pharmacological and nonpharmacological approaches. The most common approaches were sleep promotion (19.5%; 106/543), noise reduction (18.2%; 99/543) and pain treatment (17.7%; 96/543). The family was involved in *delirium* treatment in only 14.2% (77/543) (Figure 2). Measures for sleep promotion included reducing hearing with ear plugs (28.7%; 115/401), minimizing light (22.9%; 92/401) and noise (20.2%; 81/401), cognitive stimulation (14.8%; 59/401) and pharmacological therapies (13.5%; 54/401).

When analyzing the pharmacological approach to hyperactive *delirium*, physicians reported the use of haloperidol (91.5%; 107/117), quetiapine (71.8%; 84/117) and alpha 2 agonists (43.6%; 51/117) (Figure 1). Benzodiazepines were reported to be used by 23.9% (28/117). Subsyndromal *delirium*^(24,25)^ was reported by 76% of physicians (68/117) and *delirium* treatment was reported by 46.2% (54/117) of physicians.

### Point prevalence study

Twenty ICUs were invited to participate in the PPS. A total of 17 ICUs were included, from the North, Center and South of Portugal, with a total of 189 beds. We included 192 patients (37.5% females and 62.5% males). The number of patients was higher than the number of beds available since, in some cases, during the study duration, a patient was discharged from the ICU and another was admitted to the same bed (Table 3).

**Table 3:** Point prevalence study: demographic and clinical characteristics of patients

Patients included (n)	192
Patients excluded (n) Death during the study period	5
Withhold or withdraw life-sustaining decisions	2
Number of ICUs (n)/patients’ distribution (n) North	6 (35)/73 (38)
Center	2 (11.8)/14 (7.3)
South	9 (52.9)/105 (54.7)
Islands	0/0
ICUs characterization (n = 17) Mixed	16
Neurocritical	1
Total of ICU beds	189
Sex Female	72 (37.5)
Male	120 (62.5)
Severity index APACHE	19 (9.8)
SAPS II	42 (18.8)
SOFA at admission	7.3 (3.7)
SOFA in the study day	5.9 (4.1)
Type of admission Medical	118 (61.5)
Surgical	41 (21.4)
Neurocritical	14 (7.3)
Trauma	16 (8.3)
Missing values	3 (1.6)
Diagnosis on admission Septic shock	71 (37)
Respiratory failure	31 (16)
Elective surgery	19 (9.9)
Trauma	17 (8.9)
Urgent surgery	18 (9.4)
Neurologic	13 (6.8)
Cardiac arrest	7 (3.6)
Metabolic disturbs	5 (2.6)
Missing value	4 (2.0)
Renal failure	3 (1.6)
Cardiac failure	2 (1.0)
Burn patient	2 (1.0)
Invasive mechanical ventilation (nº of patients)	108 (56.3)
Duration (days)	7.9 ± 8.7

ICU - intensive care unit; APACHE - Acute Physiology and Chronic Health Evaluation; SAPS II - Simplified Acute Physiology Score II; SOFA - Sequential Organ Failure Assessment. Results presented as n (%) or mean ± standard deviation. There were some missing values.

Most of the ICUs were mixed, just one was specific for neurocritically ill patients, and the main type of admission was medical (61.5%). The mean Acute Physiology and Chronic Health Evaluation (APACHE) II and Simplified Acute Physiology Score (SAPS) II scores at admission were 19 and 42, respectively. The Sequential Organ Failure Assessment (SOFA) score at admission was 7.3 and on the study day was 5.9. Fifty-six percent of the patients were under invasive mechanical ventilation. There were some missing values in the case report form that are mentioned in the results presentation (Table 3).

### Sedation

Sedation was used in 87 patients (45.3%), with propofol being the most used drug (58.6%; 51/87), followed by midazolam (27.5%; 24/87), remifentanil (8.0%; 7/87) and dexmedetomidine (4.6%; 4/87). The mean propofol daily dose was 2400mg/day, and midazolam was 157mg/day. Apart from these 24 patients with an intravenous perfusion of midazolam, 20 other patients were also receiving the enteric benzodiazepines lorazepam, alprazolam, oxazepam and bromazepam regularly (Table 4).

**Table 4:** Point prevalence study: sedative and analgesic intravenous medication

Sedative medication	Total (n = 87)	Total dose per day (mg)^*^
Propofol	51 (58.6)	2.400 ± 1,701
Midazolam	24 (27.6)	157.2 ± 134.4
Remifentanil Remifentanil	7 (8.0)	Dexmedetomidine
Dexmedetomidine	4 (4.6)	-
Haloperidol	1 (1.2)	-
Analgesic medication	Total (n = 240)	
Acetaminophen	83 (34.6)	3.000 ± 404
Fentanyl	51 (21.3)	2.16 ± 1.6
Metamizole	39 (16.3)	4.000 ± 1300
Morphine	22 (9.2)	24 ± 31.4
Tramadol	19 (7.9)	300 ± 82.3
Remifentanil	10 (4.2)	-
Nonsteroidal anti-inflammatory	7 (2.9)	-
Ketamine	5 (2.0)	
Alfentanil	3 (1.3)	-
Dexmedetomidine	1 (0.4)	

Results presented as n (%).^*^Presented as the mean ± standard deviation, only if present in more than 10 patients.

A sedation assessment was performed in 88.5% of patients (170/192), at a minimum of 1 time per day and a maximum of 17 times/per day. The most commonly used scale was the RASS (58.2%; 99/170). The Glasgow coma scale (GCS),^(26)^ was used alone in 18.2% (31/170) and in association with the RASS scale in 15.9% (27/170) (Table 5).

**Table 5:** Point prevalence study: sedation, analgesia and *delirium* assessment tools (individual use or in combination)

Sedation assessment tools (n = 170; 88.5%) RASS	99 (58.2)
GCS	31 (18.2)
RASS + GCS	17 (10.0)
RASS + Ramsay + GCS	10 (5.9)
RASS + Ramsay	8 (4.7)
Ramsay	5 (2.9)
Analgesia assessment tools (n = 175; 91.1%) BPS	80 (45.7)
NRS	36 (20.6)
VAS	17 (9.7)
ESCID	10 (5.7)
BPS + VAS	5 (2.9)
BPS + VAS + FPS	5 (2.9)
Others less frequent combinations	22 (12.6)
*Delirium* assessment tools (n = 16; 8.3%) Clinical assessment	11 (68.8)
CAM-ICU	5 (31.3)

RASS - Richmond Agitation-Sedation Scale; GCS - Glasgow Coma Scale; BPS - Behavioral Pain Scale; NRS - Numeric Rating Scale; VAS - Visual Analog Scales; ESCID - *Escala de Conductas Indicadoras de Dolor*; FPS - Faces Pain Scale; CAM-ICU - Confusion Assessment Method for Intensive Care Unit. Multiple option response.

**Figure 3:**
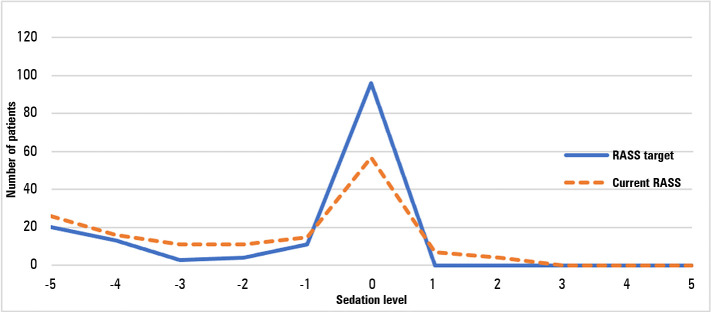
Sedation target compared with real sedation; sedation level assessed with the Richmond Agitated Sedation Scale. RASS - Richmond Agitation-Sedation Scale.

To assess the adequacy of sedation (oversedation and undersedation), we compared the sedation target for a specific day with the sedation presented by patients. Figure 3 shows the variation in sedation level, in RASS points, between the target and the measured RASS.

In 147 patients, 57% (n = 84) achieved the sedation target desired for that day, 32% (47 patients) were oversedated compared with the targets, and 11% (16 patients) were undersedated.

### Analgesia

Analgesia was used in 162 patients. Acetaminophen was the most frequently used analgesic (34.6%; 83/240) at a mean dose of 3000mg/day (SD ± 404), followed by opioids: fentanyl (21.3%; 51/240), morphine (9.2%; 22/240), tramadol (7.9%; 19/240) and remifentanil (4.2%; 10/240). The mean fentanyl dose was 2.16mg/day (SD ± 1.6), and the mean morphine dose was 24 mg/day (SD ± 31.4) (Table 4).

Acetaminophen was the most commonly used agent in monotherapy (n = 61), followed by metamizole (n = 34) and fentanyl (n = 34). The most frequent analgesic association was fentanyl with acetaminophen (n = 14), followed by morphine with acetaminophen (n = 12).

All ICUs used pain scales, and furthermore, some applied more than one scale in the same patient (Table 5). The BPS^(19)^ was the most used scale (45.7%; 80/175), followed by the Numeric Rating Scale (NRS)^(27)^ (20.6%; 36/175), VAS^(21)^ (9.7%; 17/175) and *Escala de Conductas Indicadoras de Dolor* (ESCID)^(28)^ (5.7%; 10/175).

### Delirium

*Delirium* assessment was reported in 8.3% (16/192) of the patients. In 17.7% (34/192), it was not possible to access *delirium* due to deep sedation (RASS -4 and -5), and 14% (27/192) of the patients had missing information. The majority of the patients (59.9%, 115/192) did not have any *delirium* assessment.

Concerning the 8.3% that were assessed for *delirium*, the method used had a significant percentage of missing values (91.7%). Assessment with CAM-ICU was reported only in 5 patients. Subsyndromal *delirium* was not assessed in the ICUs. Physical restraints were used in 18 patients (9.4%).

## DISCUSSION

The clinical practice reported in the survey did not accurately reflect the clinical practice reported by the PPS. Oversedation both identified in the survey and the PPS, is still present in Portuguese ICUs, with a high percentage of benzodiazepine use. *Delirium* and subsyndromal *delirium* daily assessment with validated scales was not performed even though the survey reported *delirium* assessment in more than 70%.

Analgesia with opioids was the first reported choice, but acetaminophen was the leading drug, as described in the PPS.

Use of the analgesia, sedation and *delirium* protocol was reported by 50% of the physicians, which was higher than other European surveys (31%;^(29)^ 19.4%)^(11)^ and more similar to Brazilian surveys (52.7%^(16)^; 59,5%^(9)^).

**Figure 4:**
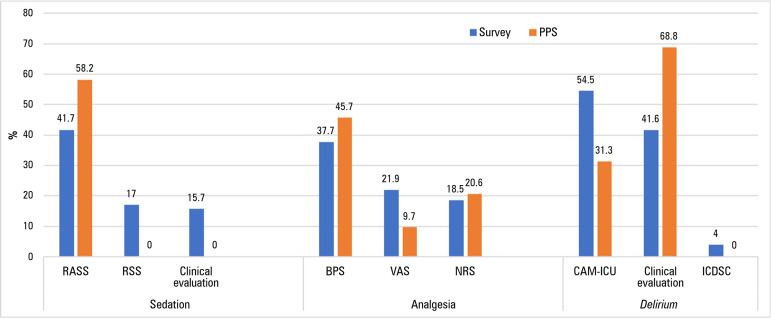
Comparison of the three main responses concerning sedation, analgesia and *delirium* assessments in the survey and point prevalence study. PPS - point prevalence study; RASS - Richmond Agitation-Sedation Scale; RSS - Ramsay Sedation Scale; BPS - Behavioral Pain Scale; VAS - Visual Analog Scales; NRS - Numeric Rating Scale; CAM-ICU - Confusion Assessment Method for Intensive Care Unit; ICDSC - Intensive Care Delirium Screening Checklist.

Daily sedation monitoring, a fundamental approach for sedation management, is a common practice in Portuguese ICUs, reported in the survey (92%) and confirmed by the PPS (88.5%). Despite this high percentage, there is still space for improvement, and an effort should be made for all patients to have access to sedation monitoring as a means to avoid oversedation and related complications. The RASS scale was the most frequently used and reported sedation scale, perhaps because of its ease of use, with positive scores for agitation and negative scores for sedation (Figure 4).

The GCS was reported in the survey as part of the sedation assessment (15.3%) and was also used in clinical practice (reported in PPS as a sedation assessment toll in 18.2%). It was not possible to determine if it was used as a sedation scale or for consciousness evaluation (our study included 7.3% of neurocritical patients). The GCS should not be used as a sedation scale because it has more inconsistent interobserver reliability, particularly in nonverbal intubated patients, compared with validated sedation scales.^(26)^ Other studies have used this scale for sedation, but it has always been pointed out as a study limitation.^(6,30)^

With the PPS, we concluded that 32% of patients were oversedated and 11% were undersedated when compared with the sedation targets defined for that specific day (Figure 3). Therefore, 57% had an adequate RASS level. Interestingly, in the survey, the percentage of oversedation reported by physicians was 46.4%, which was even higher than we observed in the PPS. This is an improvement target, as oversedation is related to worse ICU outcomes.

When we considered sedation, propofol was the most frequently reported sedative both in the survey and the PPS. Benzodiazepines, namely, midazolam, were reported in PPS in 27.5%. When comparing our results with data from other countries, this is a positive trend toward the best clinical practice, as the target should always be toward light sedation.^(31)^ Reports around Europe show differences in benzodiazepine use. For example, midazolam is more frequently used in some countries, such as Spain,^(15)^ France,^(14)^ Poland^(11)^ (respectively: 16.7%; 75%, >90% of patients), and less frequently used in the UK and Nordic countries,^(13)^ where propofol occupies the leading position (in 80-98% of cases). Dexmedetomidine has been available in Portugal since 2011 but was used in a smaller number of patients (4.6%), possibly due to cost issues or lack of experience.^(32)^

Pain assessment is common in Portuguese ICUs (the survey reports 93% and the PPS 91.1%). It should be generalized to all patients, as pain control is a main goal in ICU care. The importance of pain control is well demonstrated in the eCASH (early Confort using Analgesia, minimal Sedatives and maximal Human care)^(7)^ concept, where effective pain relief is the first priority associated with minimal sedation. Opioids remain the mainstay for pain management; however, because of safety issues (such as sedation, *delirium*, respiratory depression, ileus and immunosuppression), there is a trend toward the use of a multimodal analgesia approach. This strategy allows opioid sparing and analgesic effectiveness improvement with other agents, such as acetaminophen, ketamine and nonsteroidal anti-inflammatory drugs (NSAIDs). Nevertheless, it is important to keep in mind that the best strategies should always be individualized.

There were some differences in the first analgesic option between the survey and the PPS. In the survey, opioids were the preferred analgesic, followed by acetaminophen. In the PPS, the most frequently used analgesic medication was acetaminophen, in monotherapy or in association with opioids.

In the PPS, opioids were the second most commonly used analgesic agents. Locoregional analgesia use was still marginal in most of our ICUs.

In the PPS, all participating ICUs assessed pain with validated scales, a fundamental and priority approach for patient best care. The BPS was the most frequently used scale (45.7%; 80/175), followed by the NRS (20.6%; 36/175), the VAS (9.7%; 17/175) and the ESCID (5.7%; 10/175). Similar reports were seen in the survey, with BPS, VAS and NRS being the most used scales (Figure 4). In some studies, pain evaluation is dependent on patient collaboration, leaving noncollaborating patients without evaluation. In France, pain assessment in communicative patients was reported to occur in approximately 70% of cases and in noncommunicative cases, 30%.^(14)^ A particularity of our study was the diversity of pain scales used, with the behavioral assessment tools being preferentially listed for patients unable to self-report pain, such as BPS. This was not the focus of this study but could perhaps be explained by the difficulty in pain measurement and the attempt to choose the most reliable and valid pain assessment method for each patient.

*Delirium* assessment presented the most divergent results between the survey and the PPS. The authors believe this is one of the procedures with more opportunity for improvement and further interventions.

In the survey, physicians described *delirium* as an important cause of mortality and morbidity, and 70% reported assessing it daily (most of them using CAM-ICU).

This was not translated into practice, as with PPS, there was a small percentage of *delirium* assessment by validated scales (< 5%) and a high percentage of missing values. The CAM-ICU was the most used and reported scale in both studies (Figure 4).

Hypoactive *delirium* is frequently not identified without a routine and validated screening tool, and even hyperactive *delirium* can be undiagnosed or misdiagnosed.^(8)^ Different studies show that the use of validated scales along with education improves the ability to detect *delirium* in the ICU.^(33,34)^ We assume that the lack of a validated *delirium* assessment is responsible for the low *delirium* prevalence (7.8%), contrary to the majority of the studies with a higher prevalence of *delirium* (approximately 31.8%).^(35)^ The authors believe that there are several reasons that explain the low adherence to formal *delirium* screening. On the one hand, in our survey, more than 60% of respondents considered *delirium* diagnostic scales difficult to apply. On the other hand, and according to a survey performed by Ely et al.^(36)^ in 2001, the majority of health care practitioners believed *delirium* was a prevalent problem; nonetheless, protocols for managing *delirium* were scarce. In our survey, even though the existence of protocols for ASD was considered useful by 95% (104/110) of the physicians, fewer than 50% described having such protocols in their ICUs (Table 2).

According to PPS, physical restraints were used in 9.4% of patients. The PPS did not address the rationale for physical restraints in intubated and nonintubated critically ill patients or the type of physical restraints. There is a lack of information about its use, safety, efficacy, and outcomes in critically ill adults.^(6)^ Restraint use varies widely between 0% in some European countries and more than 75% in North America. Prior to this study, the only Portuguese information about restraint usage was 87% in the ICU.^(37)^

Our study presents some limitations. First, the time elapsed since the study took place. These study started before the publication of the 2018 Pain, Agitation/Sedation, *Delirium*, Immobility (rehabilitation/mobilization) and Sleep (PADIS) guidelines.^(6)^ At that time, the PAD-Pain, agitation/sedation and *delirium* guidelines were the main references for clinical practice.^(38)^ Second, surveys are frequently used to establish current clinical practice in a variety of health care settings; they are lowcost, quick, and easy to conduct, although achieving a high response rate and reliable information can be challenging. In this survey, there was a low participant response rate (28% response rate), similar to other surveys (French - 18.1%;^(14)^ Poland - 37,8%^(11)^). We must assume some degree of bias, as studies relying on self-report regarding clinical practices overestimate the use of evidence-based medicine compared with real-life practices.

Aside from these expected limitations, there are strengths to report: 1) To the best of our knowledge, this is the first study about analgesia, sedation and *delirium* practices in Portuguese ICUs; 2) Considering the extensive drawbacks of surveys, we also performed PPS regarding the same aspect of the survey. With the survey, we aimed to address the perception of analgesia, sedation and *delirium* practices among intensivists and with the PPS the actual practices. 3) Despite a low individual response rate in the survey, the respondents represented most of the ICUs from all the different regions of Portugal.

## CONCLUSION

To the best of our knowledge, this is the first Portuguese national study that encompasses both a national survey and a point prevalence study that provided a detailed perspective of the Portuguese approach concerning analgesia, sedation and *delirium*. This study emphasizes the need for widespread educational efforts for the implementation of evidence-based strategies for analgesia, sedation and *delirium* management in Portuguese intensive care units.
