# Erratum

**DOI:** 10.5935/0103-507X.20220020-er-en

**Published:** 2022

In the article **Sedation, analgesia, and *delirium* management in Portugal: a survey and point prevalence study**, with DOI number: 10.5935/0103-507X.20220020-en, published in the journal Revista Brasileira de Terapia Intensiva, 34(2):227-36, on page 235, after “Author contributions”:

Read:

**ACKNOWLEDGEMENTS**

Ana Albuquerque (*Hospital de S. Teotónio, Centro Hospitalar Tondela-Viseu*), Filipa Monteiro (*Hospital de Egas Moniz, Centro Hospitalar de Lisboa Ocidental*), Glória Cabral Campello (*Hospital de Portimão*), Margarida Araújo (*Hospital de Vila Franca de Xira*), Maria Eugénia Germano (*Hospital de Santo André-Leiria*), Marta Bastos (*Centro Hospitalar Vila Nova de Gaia*), Guiomar Castro (*Centro Hospitalar de São João*), Jorge Gomes (CINTESIS), Luís Bento (*Hospital de São José, Centro Hospitalar de Lisboa Central*), Raquel Nazareth (*Hospital de Loures*), Sara Gomes (*Hospital Prof Fernando da Fonseca*).

