# Supplementary material for: Sedation, analgesia, and delirium management in Portugal: a survey and point prevalence study
Source: Rev Bras Ter Intensiva. 2022 Apr-Jun;34(2):227–36. doi: 10.5935/0103-507X.20220020-en (PMC9354111; doi:10.5935/0103-507X.20220020-en)
Supplement: Supplementary file 1 [file rbti-34-02-0227-suppl01.pdf]

Sedation, analgesia, and *delirium* management in Portugal: a survey and point prevalence study

Abordagem da sedação, da analgesia e do delirium em Portugal: inquérito nacional e estudo de prevalência

Maria Carolina Paulino[1], Isabel Jesus Pereira[2], Vasco Costa[1], Aida Neves[3], Anabela Santos[4], Carla Margarida Teixeira[5], Isabel Coimbra[3], Paula Fernandes[2], Ricardo Bernardo[6], Pedro Póvoa[1], Cristina Granja[3]; on behalf of the *Grupo de Estudo de Analgesia, Sedação e Delirio of the Sociedade Portuguesa de Cuidados Intensivos/ Sociedade Portuguesa de Anestesiologia*

Appendix 1S - Survey questionnaire

SURVEY

FORMATION

- Specialty (Internal medicine / Anesthesiology / Pulmonology / Cardiology / Surgery / Nephrology / Hematology / Imunoalergology / Other)
- Subspecialty (Yes /No)
- Number of years of practice in intensive care unit - ICU (≤ 3 years / 3 - 10 years / ≥ 10 years)

(Answer options shown in parentheses, data categorized by ranges, choose only one option in each question)

ICU CHARACTERIZATION

- ICU characterization: (Mixed / Medical / Surgical / Cardiac-coronary / Neurosurgical / Other) *
- Number of beds available: (≤ 8; 8 - 12; ≥ 12) *
- Percentage of patients on invasive mechanical ventilation (< 20, 20 - 40, 40 - 70, > 70) *
- Duration of invasive mechanical ventilation: (...days) **
- Average length of stay: (... days) **

* (Answer options presented in parentheses, with data categorized by ranges, choose only one option in each question)
** (Open data, with option to place an absolute number)

CONTENT

1. In your clinical practice do you have a written protocol concerning sedation, analgesia or *delirium*? (Yes/No)
   a. If yes, which protocol do you have sedation/analgesia/*delirium*

2. Do you find protocols useful? (Yes/No)

3. Do you follow American College of Critical Care Medicine guidelines or Society of Critical Care Medicine regarding sedation, analgesia and *delirium*? (Yes/No)

4. Do you know the bundle: "ABCDEF- Awakening and Breathing Coordination, *Delirium* monitoring/management, Early exercise / mobility, and Family"? (Yes/No)

SEDATION

1. What sedation drug do you use most?
   (midazolam/diazepam/propofol/remifentanil/dexmedetomidine/fentanyl/alfentanil/morphine/haloperidol/other)
   a) If you chose "other" option, specify which: _______________

(Multiple choice answer)

2. How often do you use benzodiazepines? (< 30%, 30 - 50%; ≥ 50%)

3. Do you use any of the following sedation scales? (Ramsay / Richmond Agitation-Sedation Scale (RASS / Riker Sedation Agitation Scale)

4. In patients receiving neuromuscular blockers, how do you monitor sedation? (Sedation scales / Bispectral Index (BIS) / Other)

5. Sedation is chosen according to which criteria?
   (Age / Clinical condition / Renal function / Prediction of days of mechanical ventilation / Familiarity with the drug / Diagnosis)

(Multiple choice answer)

6. Do you define a daily sedation target? (Yes/No)

7. Do you consider that, in most of your patients, they have adequate, insufficient or excessive sedation? (Appropriate / Insufficient / Excessive)

8. Do you perform "SAB - Spontaneous Awakening Trials" daily? (Yes/No)

9. Do you perform "SBT - Spontaneous Breathing Trials" daily? (Yes/No)

Continue...

ANALGESIA

1. Do you consider pain as a frequent symptom in your ICU patients? (Yes/No)
   a) If yes, what percentage of patients are doing analgesia? ($<$ 10%, 10 - 50%, 51 - 70%, $\geq$ 70%)

2. Do you use any scale to assess pain? (Yes/No)
   a) If yes, which one do you use? (Behavioral Pain Scale (BPS) / Critical Care Pain Observation Tool (CPOT) / Faces Pain Scale / Numerical rating Pain Scale / Other)*

* (Multiple choice answer)

3. What analgesic drug do you use most?
   (Nonsteroidal anti-inflammatory drugs/Acetaminophen/Metamizol magnesium/ tramadol/morphine/remifentanil/propofol/dexmedetomidine/other) *

a. If you chose the "other" option, specify which: __________

* (Multiple choice answer)

4. Do you use preemptive analgesic medication before performing a procedure, including nursing procedures (venous punctures, naso-gastric tube placement, bed mobilization)? (Yes/No)

DELIRIUM

1. What is the *delirium* frequency in your ICU? ($<$ 10%, 10 - 25%; 26 - 50%, 51 - 70%, $\geq$ 70%)

2. How often is *delirium* in patients on invasive mechanical ventilation? ($<$ 10%, 10 - 25%; 26 - 50%, 51 - 70%, $\geq$ 70%)

3. Do you consider *delirium* as an impacting factor in any of these indicators?
   (Mortality / Morbidity / ICU cost / Duration of invasive ventilation / ICU length of stay / Length of stay/Ventilator-associated pneumonia) *

* (Multiple choice answer)

4. Is *delirium* monitoring daily? (Yes/No)

5. If yes:
   a) How is this *delirium* assessment performed? (CAM-ICU, ICDSC, Clinical evaluation, Other)
   b) Do you consider these scales easy to apply? (Yes/ No)
   c) Do you consider these scales good discriminators of patients with *delirium*? (Yes/No)
   d) Who do *delirium* assessment? (Physicians / Nurses / Both)?
   e) How many times per day is this assessment performed? (1x day / 2x day / 3x day / $>$ 3x day / Not applicable)
   f) Have you considered the diagnosis of *delirium* in patients whose scale did not classify in this way (subsyndromal *delirium*)? (Yes/No)
   g) e) Do you consider subsyndromal delirium as an individual disease or an early stage of delirium? (Individual disease / Pre-delirium stage)
   h) Do you treat subsyndromal delirium? (Yes/No)

6. Do you carry out etiological investigation of delirium? (Yes/No)

7. Do you perform pharmacological delirium treatment? (Yes/No)

8. From the following list of drugs, choose three that you most prescribe in hyperactive delirium: (Propofol/ Benzodiazepines/ Quetiapine/ Risperidone/ Haloperidol/ Other)

9. How do you use haloperidol in hyperactive *delirium*?
   (Single preventive dose / Dose at a defined interval / Increasing bolus doses / Increasing dose in continuous infusion)

10. Do you apply any measures to promote sleep? (Yes/No)
    a) If yes, which ones? (Pharmacological / Behavioral / Earplugs / Light reduction / Noise reduction)

* (Multiple choice answer)

11. How long, on average, is family visits duration?
    ($\leq$ 30 minutes / $>$ 30 minutes and $\leq$ 1 hour / $>$ 1hour and $\leq$ 2 hours / $>$ 2 hours)

12. Is the family involved in *delirium* treatment? (Yes/No)

13. Do you use any of these strategies to promote orientation and avoid *delirium*?
    (Promote hearing or vision aids use / Use of clocks / Use of calendars)

* (Multiple choice answer)

14. Do you prescribe early mobilization? (Yes/No)

15. When the clinical situation allows it, do you do early mobilization in mechanically ventilated patients? (Yes/No)

16. Do you assess muscle strength (Yes/No)
    a) If yes, do you use any ICU mobility scale? Surgical ICU Optimal Mobilization Score (SOMS) / Perme Intensive Care Unit Mobility Score (PFIT) / Chelsea Critical Care Physical Assessment Tool (CPAx) / Functional Status Score for the Intensive Care Unit (FSS-ICU))?

17. Regarding physiotherapy:
    a) How many physiotherapists do you have in your ICU? (Choose a number) **
    b) What is the physiotherapist / patient ratio? (Choose a number, various options) **
    c) How many days a week do you have physiotherapy available? (1 ... 7) **
    d) How many hours per day is the physiotherapist in your ICU Unit? (1 hour ... 24 hours) **

** (Open data, with option to place an absolute number)

Appendix 2S - Point prevalence study questionnaire

POINT PREVALENCE STUDY- SadDay
(CRF- Case Report Form)

1. Intensive care unit (ICU) characterization

a) ICU type:

Mixed___ / Medical ___ / Surgical ___ / Cardiac-coronary ___ / Neurosurgical ___

b) Geographic location

North ___ / Center ___ / Sul ___

c) Number of patients in ICU_____

d) Do you have a written protocol for sedation, analgesia and *delirium* assessment?

Yes ____ / No ____

e) What is the physiotherapist / patient ratio? _________ (Example 1:2 / 1: 3 / 1: 4)

2. Patient characterization; Identification number ____

a) Patient data:

a.1) Patient profile: Intensive (level III) ____ / Intermediate (level II) ____

a.2) Gender: F ____ / M ____

a.3) APACHE II ____ / SAPS II ____

a.4) Admission SOFA: ____ / SOFA on the day study____

a.5) Type of admission: Elective ____ / Urgent ____

a.6) Characterization of admission: Medical ___ / Surgical ___ / Coronary ___ / Trauma ___ / Neurological ___ / Neurosurgical ___

a.7) Hospital admission date: ___ / ___ / ___

a.8) ICU admission date: ___ / ___ / ___

a.9) Diagnosis on ICU admission (you can choose more than one option):

1) Infectious disease _____
2) Respiratory failure _____
3) Renal dysfunction _____
4) Septic shock _____
5) Metabolic disorders _____
6) Cardiorespiratory arrest _____
7) Trauma _____
8) Urgent surgery _____
9) Elective surgery _____

a.10) Invasive mechanical ventilation: Yes ____ / No ____

If yes, number of days of invasive mechanical ventilation _____

b) Sedation

b.1) Is the patient sedated: yes _______ / no _______

b.1.2) If yes, what drugs are used for sedation?

Propofol __ / Remifentanil ___ / Midazolam ___ / Diazepam ___ / Lorazepam ___ / Dexmedetomidine ___ / Haloperidol ___ / Others ___

b.2) Is the patient taking benzodiazepines: Yes ____ / No ____

b.2.1) If yes, what is the administration route: Oral SOS ___ / Oral fixed rate____ /

IV SOS ___ / IV bolus___ / IV continuous infusion ___

Continue...

b.3) Drugs in continuous infusion:

	Sedative name	Maximum dose (mg/h)	Route of administration	Total dose (mg)
1				
2				
3				
4				

b.4) Drugs in bolus:

	Sedative name	Route of administration	Total dose (mg)
1			
2			
3			
4			

b.5) Do you use scales to assess sedation? Yes _____ / No _____

b.5.1) If yes, which scales are used: RASS _____ / GCS _____ / SAS ____ / Ramsay____

b.5.2) Who applies the scales? Physician _____ / Nurse _____ / Other _____

b.5.3) How many times a day was this assessment performed? ______ (Absolute number)

b.6) Is the patient under neuromuscular blocker? Yes _____ / No _____

b.6.1) If yes, is sedation monitoring being performed with BIS? Yes ____ / No_____

b.7) What is the target RASS for the patient?

-5 ___ / - 4 ___ / -3 ___ / -2 ___ / -1 ___ / 0 ___ / +1 ___ / +2 ___ / +3 ___ / +4 ___

b.8) What is the patient's current RASS?

-5 ___ / -4 ___ / - 3 ___ / -2 ___ / -1 ___ / 0 ___ / +1 ___ / +2 ___ / +3 ___ / +4 ___

c) Analgesia

c.1) Is the patient doing analgesia? Yes____ / No _____

c.2) What is the analgesic used? Morphine /Fentanyl / Alfentanil/ Remifentanil / Ketamine/ Nonsteroidal anti-inflammatory / Acetaminophen / metamizole magnesium/Other

c.3) Are you undergoing multimodal analgesia? Yes____ / No _____

c.4) What is the dosage?

Oral SOS ____ / Fixed oral ____ / IV SOS ____ / Fixed IV / ____ / IV perfusion ____

c.5) Drugs in continuous infusion:

	Analgesic	Maximum dose (mg/h)	Route of administration	Total dose (mg)
1				
2				
3				
4				

c.6) Drugs in bolus:

	Analgesic	Route of administration	Total dose (mg)
1			
2			
3			

c.7) Is the patient with loco-regional analgesia? Yes____ / No ____

c.8) How is pain assessment performed? BPS scales ___ / CPOT ___ / VRS ___ / NRS ___ / VAS ___ / Face scale ____ / None ____ / Other ____

c.9) How makes pain assessment? Physician ____ / Nurse ____

c.10) Is analgesia prescribed prior to procedures, dressings, or other situations that trigger pain (preemptive)? Yes____ / No____

d) *Delirium*

Complete only if the RASS is greater than or equal to - 3

d.1) Does the patient have *delirium*? Yes ____ / No ____

Continue...

...Continuation

If yes,

d.1.1) What is *delirium* type? Hyperactive / Hypoactive / Mixed

d.1.2) How is *delirium* assessment done? Clinical evaluation / CAM-ICU / ICDSC / Other

d.1.3) Who made this assessment? Physician _____ / Nurse_____

d.1.4) If you used scales, how many evaluations were carried out in the last 24 hours? ____ (absolute number)

d.1.5) What medication was prescribed in *delirium* patient?

Haloperidol / Dexmedetomidine / Propofol / Midazolam / Another benzodiazepine / Other

d.1.6) Is the family involved in *delirium* treatment? Yes ____ / No ____

If no,

d.1.7) Does de patient has subsyndromal delirium? Yes ____ / No ____

d.1.8) How was subsyndromal delirium assessment done?

Clinical evaluation / CAM-ICU / ICDSC / Other

d.1.9) Who made this assessment? Physician / Nurse

d.1.10) If you used scales, how many evaluations were carried out in the last 24 hours? ____

d.2) Has the patient been medicated with drugs to promote sleep? Yes ____ / No ____

d.3) In the last 24h witch strategies were used to promote patient orientation?

Reorientation ____ / Prosthesis (auditory, visual ...) ____ / Clocks ____ / Calendars ___ / Other ____

d.4) The patient was restraint physical in the past 24 hours, including during nighttime?

Yes ____ / No ____

d.5) Early mobility was applied? Yes ____ / No ____

d.6) In the last 24 hours did the patient had physiotherapy? Yes ____ / No ____
